# An Occupational Job Strain Index based on five Norwegian nationwide surveys of living conditions on work environment

**DOI:** 10.1186/s13104-026-07754-1

**Published:** 2026-02-28

**Authors:** Åsmund Hermansen, Giang Huong Le

**Affiliations:** 1 Department of Social Work, Child Welfare and Social Policy, Faculty of Social Sciences, OsloMet-Oslo Metropolitan University, Oslo, Norway; 2 National Institute of Occupational Health (STAMI), Oslo, Norway

**Keywords:** Occupational Job Strain Index, Job Exposure Matrix, Register data, Working conditions, Psychosocial job exposure

## Abstract

**Objectives:**

This paper aims to provide comprehensive documentation of the Occupational Job Strain Index (JSI), developed using data from the Norwegian nationwide Survey of Living Conditions conducted in 2006, 2009, 2013, 2016, and 2019. The JSI, based on Karasek’s Demand-Control Model, offers detailed information on working conditions, addressing a gap in national register data. The surveys, including information on the psychosocial working environment, contributed to the development of the JSI through self-reported information and psychosocial exposure items by Statistics Norway (SSB).

**Data description:**

This paper includes the scripts documenting the construction of the Psychosocial Job Exposure Matrix and the Occupational Job Strain Index (Data File 1), along with a script containing the key for converting 4-digit STYRK-08 codes into 4-digit STYRK-98 codes and the procedure for merging the index with register data (Data File 2). Data File 3 contains the complete Psychosocial Job Exposure Matrix and Occupational Job Strain Index. Data File 4 includes a full codebook.

## Objective

National register data have long been a valued resource for research in the Nordic countries, providing extensive longitudinal data on the entire population across a myriad of life domains [1]. However, these data sets often lack detailed information on working conditions. To address this, researchers have developed job exposure matrices (JEMs) that provide work environment information based on job titles [2]. JEMs have proven useful in research, dating back to their establishment in the 1980s, and have been developed and applied in many countries [3–5]. Scholars in Nordic countries have also developed and evaluated national JEMs [6–8].

In a previous study, Le, Hermansen and Dahl [9] constructed and validated a Job Strain Index (JSI) based on five surveys from the Norwegian nationwide Survey of Living Conditions conducted in 2006, 2009, 2013, 2016, and 2019, with a total sample of 43,977 individuals. Job strain was composed of items belonging to the two dimensions of Karasek’s Demand-Control model, job demands and job control (1979) [10]. The measurement of psychological demands and job control followed the guidance of the General Nordic Questionnaire (QPSNordic) [11]. In our study, psychological job demand was measured by four items: (1) quantitative demands, (2) conflicting ways of doing things, (3) insufficient resources, and (4) contradictory requests. Job control or decision-latitude was measured by six items: (1) decide how to go about the work, (2) decide the pace of work, (3) make important decisions, (4) use skills, (5) develop skills, and (6) monotonous work.

This paper aims to provide comprehensive documentation and access to the Occupational Job Strain Index, enabling researchers to employ the index in register-based research. It also includes access to the necessary scripts demonstrating how these exposures were calculated, along with the completed Job Strain Index drawn from all five surveys. These scripts are available in Stata format. The index can be merged with register data using occupational codes (STYRK-98) and gender.

## Data description

The Job Exposure Matrix (JEM) was constructed using data obtained from the Norwegian nationwide Survey of Living Conditions on work environment, conducted in 2006, 2009, 2013, 2016, and 2019. The surveys were conducted by Statistics Norway using Computer Aided Telephone Interviewing. The samples for each survey were randomly selected from the population aged 18–69 years, representing the active workforce in Norway. The response rates ranged from a high of 67.2% in 2006 to a low of 52.6% in 2016.

The surveys encompassed questions related to the psychosocial working environment. Our study’s Job Strain Index (JSI) is based on self-reported information, with items measuring psychosocial exposures developed by Statistics Norway (SSB). In line with Karasek’s Demand-Control Model, the index merges the psychological demand index (job demand) and decision-latitude index (job control). Psychological demands and job control measurements adhered to the General Nordic Questionnaire (QPSNordic) guidelines.

While our JSI construction is inspired by Karasek’s Demand/Control model (1979), we used only ten items to measure psychosocial work exposures, covering the dimensions of job demand and job control. This approach is different from Karasek’s original Job Content Questionnaire [12], which comprises 49 items addressing multiple factors. Therefore, our JSI represents a shorter version of the JCQ, closely aligning with the Swedish version [13].

To construct a Psychosocial Job Exposure Matrix, each item was dichotomized following the same procedure as previous Norwegian study [7], splitting each scale at the median to identify those who are exposed vs. non-exposed. For the individual exposures, we calculated the median value for each item using the raw values and then used the median as a cut-off as to identify the exposed versus non-exposed individuals based on the individual information. In example for “Quantitative demands” the median value on the five-point scale was 2. Thus, those with a value above 2 (Daily = 5, a few days a week = 4, once a week = 3) were defined as “exposed”. Whereas for the occupation-based exposures, we calculated the share of exposed individuals for each item within each JEM group and used the median as a cut-off as to identify individuals defined as exposed and non-exposed based on their occupational code.

For each of these occupation-based exposures, the share of exposed individuals for each item within 268 JEM groups was calculated, providing the overall share of exposed within a total of 333 unique occupational codes. This implies that occupational codes with a value of 0 on one of the exposures implies that none with these occupational codes, belonging to the same JEM-group, has provided an answer that involves exposure. In contrast, the value 100 means that all respondents with that occupational code, belonging to the same JEM-group, have provided an answer that involves exposure. For a detailed description regarding the construction of the Psychosocial Job Exposure Matrix please see Le, Hermansen and Dahl [9, p. 3–9].

Table 1 below includes the scripts documenting the construction of the Psychosocial Job Exposure Matrix and the Occupational Job Strain Index (Data File 1), along with a script containing the key for converting 4-digit STYRK-08 codes into 4-digit STYRK-98 codes and the procedure for merging the index with register data (Data File 2). Data File 3 contains the complete Psychosocial Job Exposure Matrix and Occupational Job Strain Index. Data File 4 includes a full codebook. Please note that Data file 1 and Data file 2 are found under “Related documents and other materials” in the data repository.

**Table 1:** Overview of data files

Label	Name of data file/data set	File types (file extension)	Data repository and identifier (DOI or accession number)
Data file 1	Datafile1_Constructing_Job_Strain_Index.do	Stata script file (.do)	Sikt –Norwegian Agency for Shared Services in Education and Research (10.18712/NSD-NSD3319-V5) [14]
Data file 2	Datafile2_Merging_Psychosocial_exposure_index.do	Stata script file (.do)	Sikt –Norwegian Agency for Shared Services in Education and Research (10.18712/NSD-NSD3319-V5) [14]
Data file 3	NSD3319.dta	Stata data file (.dta)	Sikt –Norwegian Agency for Shared Services in Education and Research (10.18712/NSD-NSD3319-V5) [14]
Data file 4	NSD3319 codebook.html	HyperText Markup Language (.html)	Sikt –Norwegian Agency for Shared Services in Education and Research (10.18712/NSD-NSD3319-V5) [14]

## Limitations

- The developed matrix and the Occupational Job Strain Index are tailored for analyses based on Norwegian register data, which restricts their wider use.
- There is no official key of correspondence between the 4-digit STYRK-98 and the 4-digit STYRK-08 codes, leading to potential challenges when aligning or merging datasets that use different versions of the STYRK classification system. This discrepancy may introduce errors or inconsistencies.
- The JEMs were derived from individual exposure measurements, which could result in assignment errors due to imprecise exposure information for each job and other errors in job coding and duration. The JEMs assign the same exposure estimates to all workers with identical job titles, which may affect inter-individual variability, particularly among workers with specific tasks.
- This approach may introduce a subjective bias and the risk of false positive results, as it reflects the individual perception of work exposure and health outcomes. For instance, workers with health problems might report higher degrees of psychosocial exposures than healthy workers, skewing the results.
- While STYRK (Standard for Norwegian Occupational Classification) is based on the International Standard Classification of Occupations (ISCO), there are Norway-specific adaptations at the detailed digit level. Researchers wishing to apply this JEM in international comparative studies would need to develop crosswalks between STYRK and ISCO codes.
- The current JEM represents a fixed reference point based on surveys from 2006 to 2019. Any future updates incorporating additional survey waves would need to document potential changes in median thresholds and their impact on exposure classification.

## Data Availability

The data described in this Data note can be freely and openly accessed through Sikt (Norwegian Agency for Shared Services in Education and Research) under https://doi.org/10.18712/NSD-NSD3319-V5. Please see Table 1 and reference [14] for details and links to the files and data. Note that Sikt (Norwegian Agency for Shared Services in Education and Research) requires users to login before accessing the data.

## Abbreviations

JEM: Job Exposure matrix
STYRK: Norwegian Occupational Catalogue
STYRK-98: Standard for occupational classification used from 1998
STYRK-08: Standard for occupational classification used from 2008
